# Supplementary material for: Impacts of social distancing policies on mobility and COVID-19 case growth in the US
Source: Nat Commun. 2021 May 25;12:3118. doi: 10.1038/s41467-021-23404-5 (PMC8149701; doi:10.1038/s41467-021-23404-5)

SUPPLEMENTARY INFORMATION

Impacts of Social Distancing Policies on Mobility and COVID-19 Case Growth in the US

Gregory A. Wellenius,[1,2] Swapnil Vispute,[1] Valeria Espinosa,[1] Alex Fabrikant,[1] Thomas C. Tsai,[3,4] Jonathan Hennessy,[1] Andrew Dai,[1] Brian Williams,[1] Krishna Gadepalli,[1] Adam Boulanger,[1] Adam Pearce,[1] Chaitanya Kamath,[1] Arran Schlosberg,[1] Catherine Bendebury,[1] Chinmoy Mandayam,[1] Charlotte Stanton,[1] Shailesh Bavadekar,[1] Christopher Pluntke,[1] Damien Desfontaines,[1,5] Benjamin H. Jacobson,[4] Zan Armstrong,[1] Bryant Gipson,[1] Royce Wilson,[1] Andrew Widdowson,[1] Katherine Chou,[1] Andrew Oplinger,[1] Tomer Shekel,[1] Ashish K. Jha,[4,6] Evgeniy Gabrilovich*,[1]

[1] Google, Inc. Mountain View, CA. [2] Department of Environmental Health, Boston University School of Public Health, Boston, MA. [3] Department of Surgery, Brigham and Women's Hospital and Harvard Medical School, Boston, MA. [4] Department of Health Policy and Management, Harvard T.H. Chan School of Public Health, Boston, MA. [5] ETH Zurich, Switzerland. [6] Brown University School of Public Health, Providence, RI. * email: gabr@google.com

These authors contributed equally: Wellenius, Vispute, Espinosa, Fabrikant

SUPPLEMENTARY METHODS

We performed a sensitivity analysis to assess the distribution of each outcome metric under the null hypothesis of the policies having no effect. We interpret the relative change across two periods of time (period 1: January 27-February 2, 2020; period 2: February 5-11, 2020), prior to the enactment of any state-level social distancing measures, as observations under the null hypothesis. During this time there were only 5 reported cases of COVID-19 in the U.S., which were isolated in Washington state. Although there was a small drop in the time spent away from the place of residence across these pre-intervention periods, the much larger effects on mobility observed after each of the policies suggests that our observed effect during the exposure period is in fact related to the implementation of social distancing (Supplementary Fig. 2 and 3).

Supplementary Figure 2 and 3 can be compared to Figures 2 and 3 in the main text to gain a sense of how different these effects look when the first social-distancing measures were implemented. Under the null hypothesis of no policy-affect, the median observed change is slightly below -2%. However, during the first social-distancing measures, the median drop is nearly 10 times as large at -19%. For further context, all but two counties during the first social-distancing measures are below the null distribution median, and over 96% of counties are below the null distribution 2.5% percentile. A comparison between the changes in mobility during first social-distancing measures and changes in mobility under the null hypothesis for each metric is presented in Supplementary Tables 2 and 3.

Our main analyses estimate the incremental mobility changes after versus before each of three waves of policy orders (Figure 2). However, it is also of interest to quantify the overall effect of social distancing by comparing mobility at the end versus the start of March. We define the overall effect as the change in mobility from the week before the term “social distancing” started increasing in Google Search (March 1-7th) to the last week available in the data (March 23-29th). We see a significant decrease in all metrics. The ordering of the magnitudes is comparable to those in Figure 2b. As expected, the overall magnitudes of the drops are larger than any of the incremental effects in Figure 2.

SUPPLEMENTARY TABLES

Supplementary Table 1: ANOVA sum of squares decomposition of the variation in the relative change on average time spent away from places of residence across counties for the linear model with the relative pre-post difference in time spent away from the residence as the outcome and state as the only independent variable. The results show that approximately half of the variance in the outcome is explained by differences between states and the remaining variance is largely explained by differences across counties within states.

	Sums of Squares	Degrees of Freedom	F statistic	P value	% of variance explained by each source
State	5.48	49	55.24	< 0.0001	49.76%
Residual	5.53	2733			50.24%
Total	11.01	2782			

Supplementary Table 2: Relative changes in national averages from January 27-February 2, 2020 to February 5-11, 2020 for all metrics of interest. Since these periods occurred before any orders were issued, we interpret these changes as observations under the null hypothesis of no policy effect. Although most metrics show a statistically significant decrease, the magnitude of the effect is considerably smaller compared with the policy intervention effects. The effect of the first social distancing order on time spent away from places of residence is ~12x larger. Similar differences are observed for the other metrics, ranging from ~2x to ~90x for Parks and Transit, respectively. The same reference null distribution can be used for the effects of the other two waves (i.e., emergency declaration and shelter in place).

	Effect of 1st SD Order			Pseudo Effect		
	Estimate	SE		Estimate	SE	Ratio of estimates
Time Spent Away from Residences	-24.47%	0.10%		-2.10%	0.11%	11.64
Visits to Grocery & Pharmacy	-9.27%	0.12%		-1.43%	0.14%	6.46
Visits to Parks	-10.54%	0.41%		-5.96%	0.50%	1.77
Visits to Retail & Recreation	-33.04%	0.12%		-0.66%	0.14%	49.75
Visits to Transit Stops	-22.43%	0.15%		-0.25%	0.18%	89.58
Visits to Workplaces	-27.92%	0.21%		-1.16%	0.23%	24.16

Supplementary Table 3: Distribution of relative changes across counties for metrics from January 27-February 2, 2020 to February 5-11, 2020 compared to the analogous distributions during the first social distancing measures. This table depicts the heterogeneity of the changes before any orders were issued and are interpreted as observations under the null hypothesis of no policy effect. In this table, we see that all the medians for the first social distancing measure are substantially lower than the medians under the null. Furthermore, the spread of the effects for the first social distancing measure is larger (e.g., for workplace visits the 90% range is 26 percentage points during the treatment period vs 10 percentage points under the null). The same reference null distribution can be used for the effects of the other two waves of policy interventions.

	First SD measures						Before any order				
	5 %ile	25 %ile	50 %ile	75 %ile	95 %ile		5% ile	25 %ile	50 %ile	75 %ile	95 %ile
Time Spent Away from Residences	-31%	-23%	-19%	-15%	-10%		-8%	-4%	-2%	-1%	1%
Visits to Retail & Recreation	-48%	-37%	-30%	-24%	-12%		-10%	-4%	-1%	1%	6%
Visits to Workplaces	-35%	-26%	-21%	-16%	-9%		-8%	-3%	-1%	0%	2%
Visits to Transit Stops	-42%	-26%	-17%	-9%	1%		-9%	-3%	0%	2%	9%
Visits to Parks	-37%	-22%	-10%	3%	30%		-26%	-14%	-6%	2%	19%
Visits to Grocery & Pharmacy	-22%	-13%	-6%	1%	13%		-7%	-3%	-1%	1%	7%

Supplementary Table 4: Percent change (and 95% confidence interval) in COVID-19 case growth associated with a week-on-week mobility change of a specified magnitude.

	Percent Change (95% confidence interval)		
Mobility change	2 Week lag	3 week lag	4 week lag
-5%	-9.2 (-7.3, -11.0)	-20.9 (-19.4, -22.3)	-13.0 (-9.3, -16.6)
-10%	-17.5 (-14.1, -20.9)	-37.4 (-35.0, -39.6)	-24.4 (-17.8, -30.4)
-15%	-25.1 (-20.3, -29.6)	-50.4 (-47.6, -53.1)	-34.2 (-25.4, -41.9)
-20%	-32.0 (-26.1, -37.4)	-60.8 (-57.8, -63.5)	-42.8 (-32.4, -51.6)

SUPPLEMENTARY FIGURES

Supplementary Fig. 1: Effect of first social distancing order on visits to places of work (a), visits to grocery stores and pharmacies (b), visits to retail, recreation, and eateries (c), visits to transit stops (d), and visits to parks (e). Boxplots indicate the 25th and 75th percentiles (box extent) and the median (center line of each box) of county-specific changes. The whiskers extend from the hinge to the largest value no further than 1.5 * interquartile range from the hinge. Dots represent outliers beyond the whiskers. N=2810 counties in 50 US states and Washington, DC.

a.

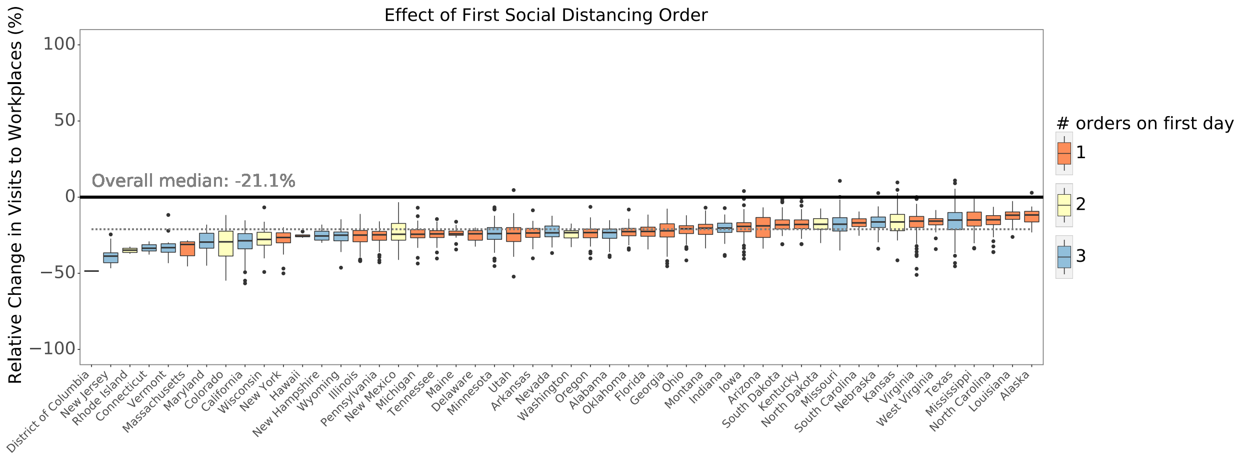

b.

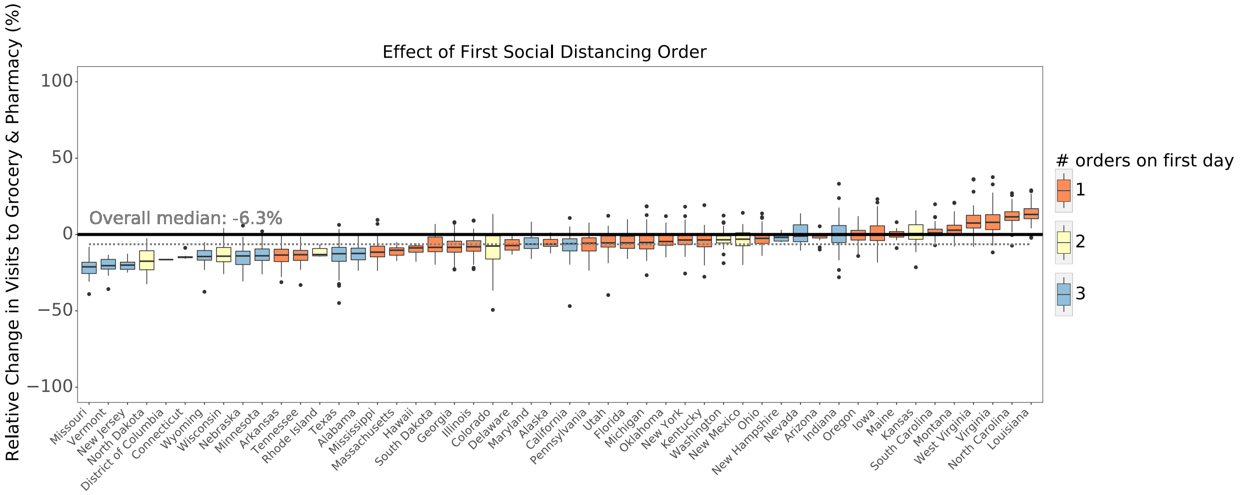

c.

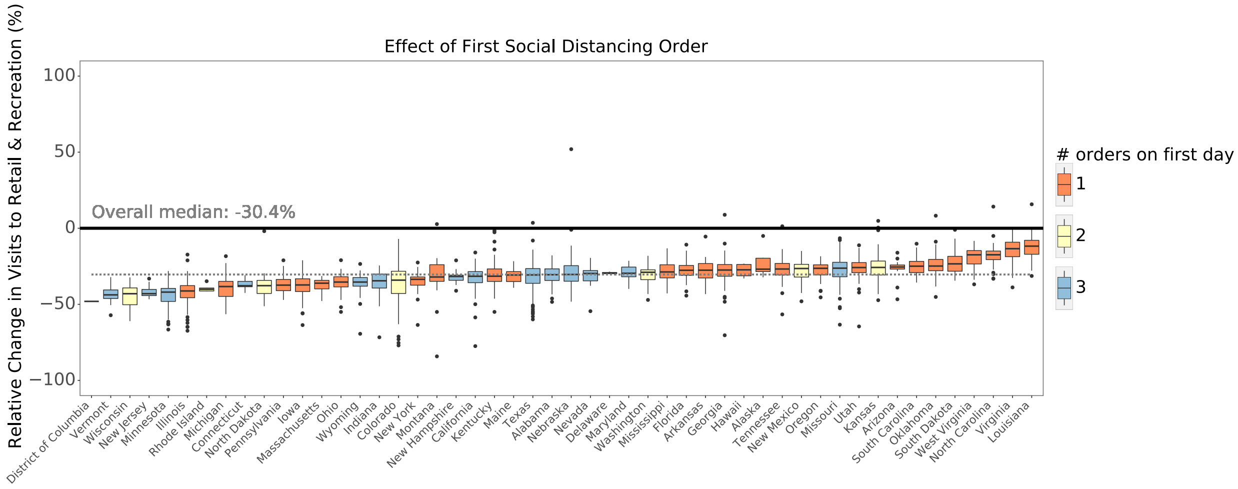
Effect of First Social Distancing Order
Relative Change in Visits to Retail & Recreation (%)
100
50
0
−50
−100
Overall median: -30.4%
orders on first day
1
2
3
District of Columbia
Vermont
Wisconsin
New Jersey
Minnesota
Illinois
Rhode Island
Michigan
Connecticut
North Dakota
Pennsylvania
Iowa
Massachusetts
Ohio
Wyoming
Indiana
Colorado
New York
Montana
New Hampshire
California
Kentucky
Maine
Texas
Alabama
Nebraska
Nevada
Delaware
Maryland
Washington
Mississippi
Florida
Arkansas
Georgia
Hawaii
Alaska
Tennessee
New Mexico
Oregon
Missouri
Utah
Kansas
Arizona
South Carolina
Oklahoma
South Dakota
West Virginia
North Carolina
Virginia
Louisiana

d.

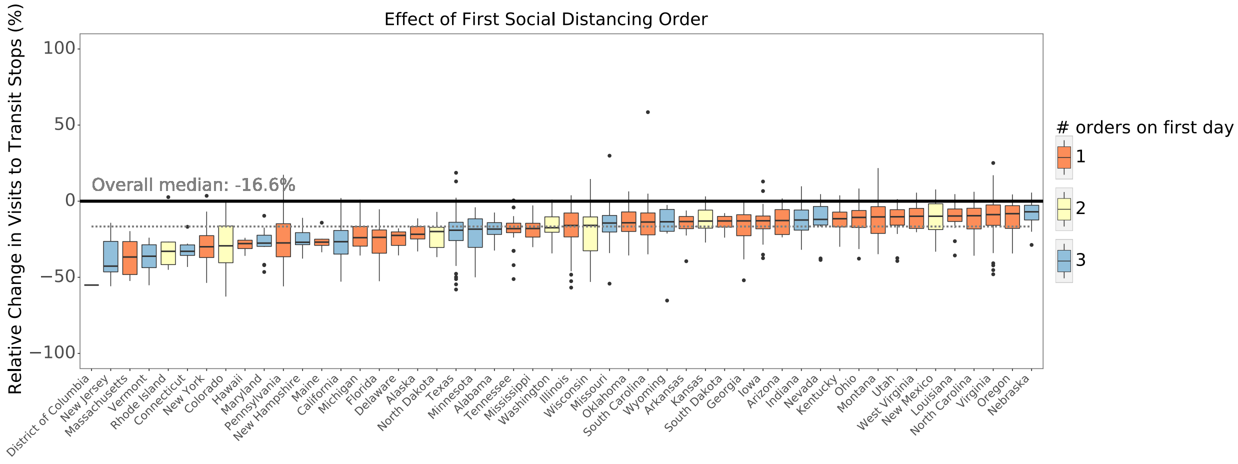
Effect of First Social Distancing Order
Relative Change in Visits to Transit Stops (%)
100
50
0
−50
−100
Overall median: -16.6%
orders on first day
1
2
3
District of Columbia
New Jersey
Massachusetts
Vermont
Rhode Island
Connecticut
New York
Colorado
Hawaii
Maryland
Pennsylvania
New Hampshire
Maine
California
Michigan
Florida
Delaware
Alaska
North Dakota
Texas
Minnesota
Alabama
Tennessee
Mississippi
Washington
Illinois
Wisconsin
Missouri
Oklahoma
South Carolina
Wyoming
Arkansas
Kansas
South Dakota
Georgia
Iowa
Arizona
Indiana
Nevada
Kentucky
Ohio
Montana
Utah
West Virginia
New Mexico
Louisiana
North Carolina
Virginia
Oregon
Nebraska

e.

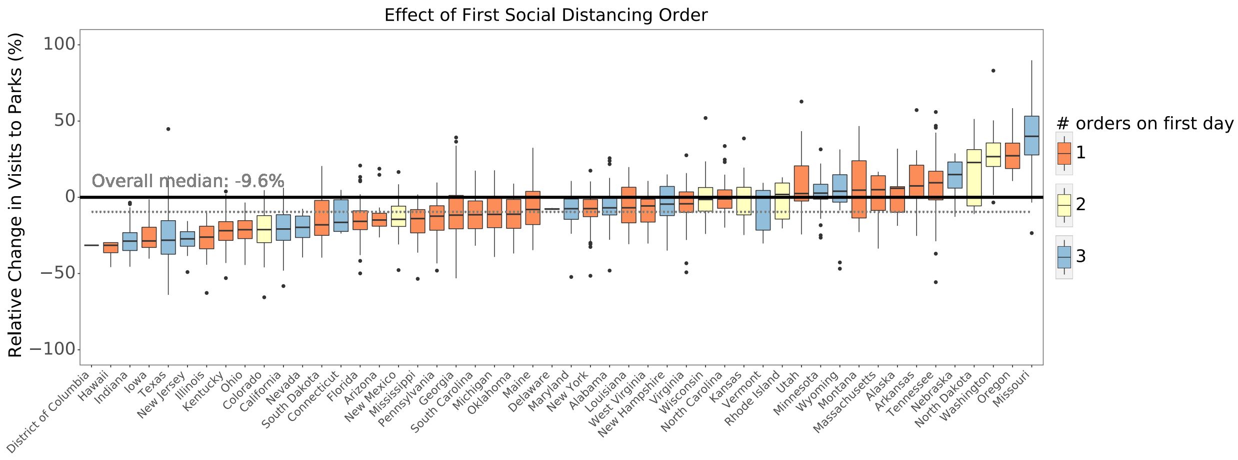

Effect of First Social Distancing Order
Relative Change in Visits to Parks (%)
100
50
0
−50
−100
Overall median: -9.6%
orders on first day
1
2
3
District of Columbia
Hawaii
Indiana
Iowa
Texas
New Jersey
Illinois
Kentucky
Ohio
Colorado
California
Nevada
South Dakota
Connecticut
Florida
Arizona
New Mexico
Mississippi
Pennsylvania
Georgia
South Carolina
Michigan
Oklahoma
Maine
Delaware
Maryland
New York
Alabama
Louisiana
West Virginia
New Hampshire
Virginia
Wisconsin
North Carolina
Kansas
Vermont
Rhode Island
Utah
Minnesota
Wyoming
Montana
Massachusetts
Alaska
Arkansas
Tennessee
Nebraska
North Dakota
Washington
Oregon
Missouri

Supplementary Fig. 2: Relative changes in national averages from January 27-February 2, 2020 to February 5-11, 2020 for all metrics of interest. The results are also shown in Supplementary Table 2. This plot can serve as a reference point for Figure 2 in the sense that these periods occurred before any orders were issued and can be interpreted as observations under the null hypothesis of no effect of policy interventions. Each bar reflects the mean and 95% confidence interval. N=2810 counties in 50 US states and Washington, DC.

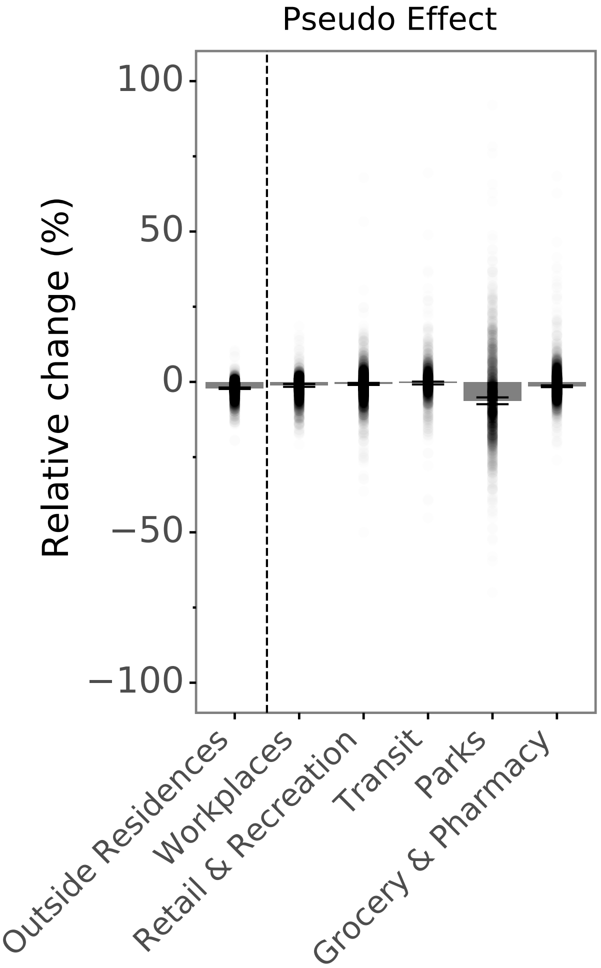

Supplementary Fig. 3: Relative changes in county averages from January 27-February 2, 2020 to February 5-11, 2020 for time spent away from the residence. Counties are grouped by state to show the heterogeneity across counties and states before any orders were issued. The changes can be interpreted as observations under the null hypothesis of no policy effect because these periods occurred before any orders were issued. Boxplots indicate the 25th and 75th percentiles (box extent) and the median (center line of each box) of county-specific changes. The whiskers extend from the hinge to the largest value no further than 1.5 * interquartile range from the hinge. Dots denote outliers beyond the whiskers. N=2810 counties in 50 US states and Washington, DC.

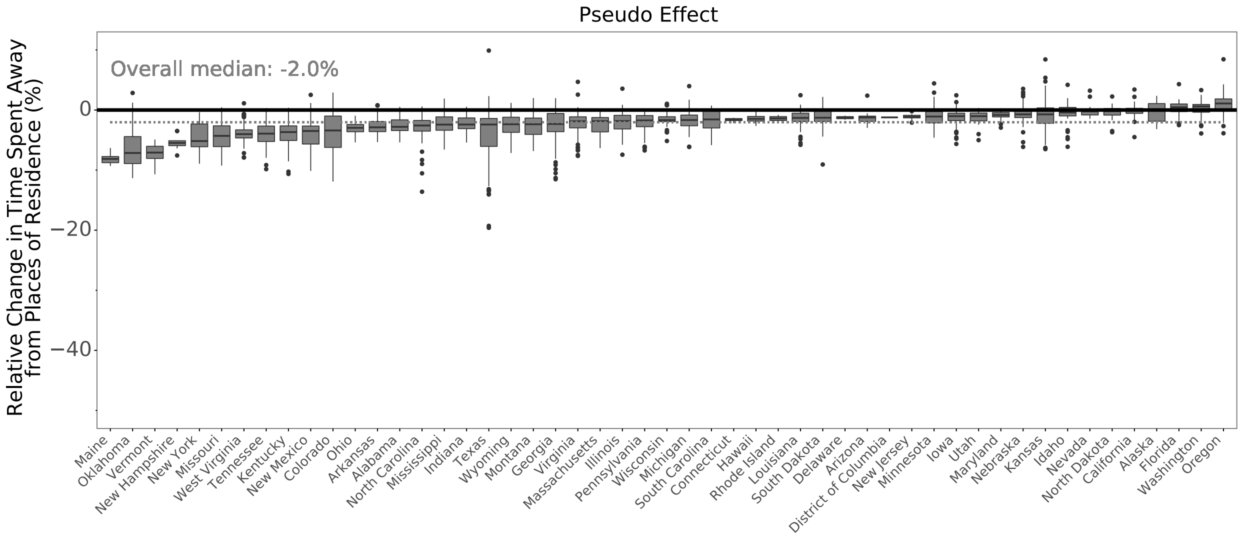

Supplementary Fig. 4: Timeline of change in average time spent away from places of residence in King County, Washington (i.e. Seattle area) (a), Westchester County, New York (b), New York County, New York (i.e., Manhattan) (c), and Santa Clara County, California (i.e. San Jose area)(d). Colored boxes denote the declaration of a state of emergency, the implementation of the first county-level social distancing order, the implementation of the first state-level social distancing order, and county and/or state-level orders for residents to shelter in place. The height of each box corresponds to the change in average time Location History users spent away from places of residence in the week before (plus a 2-day washout period) versus the week after each policy date.

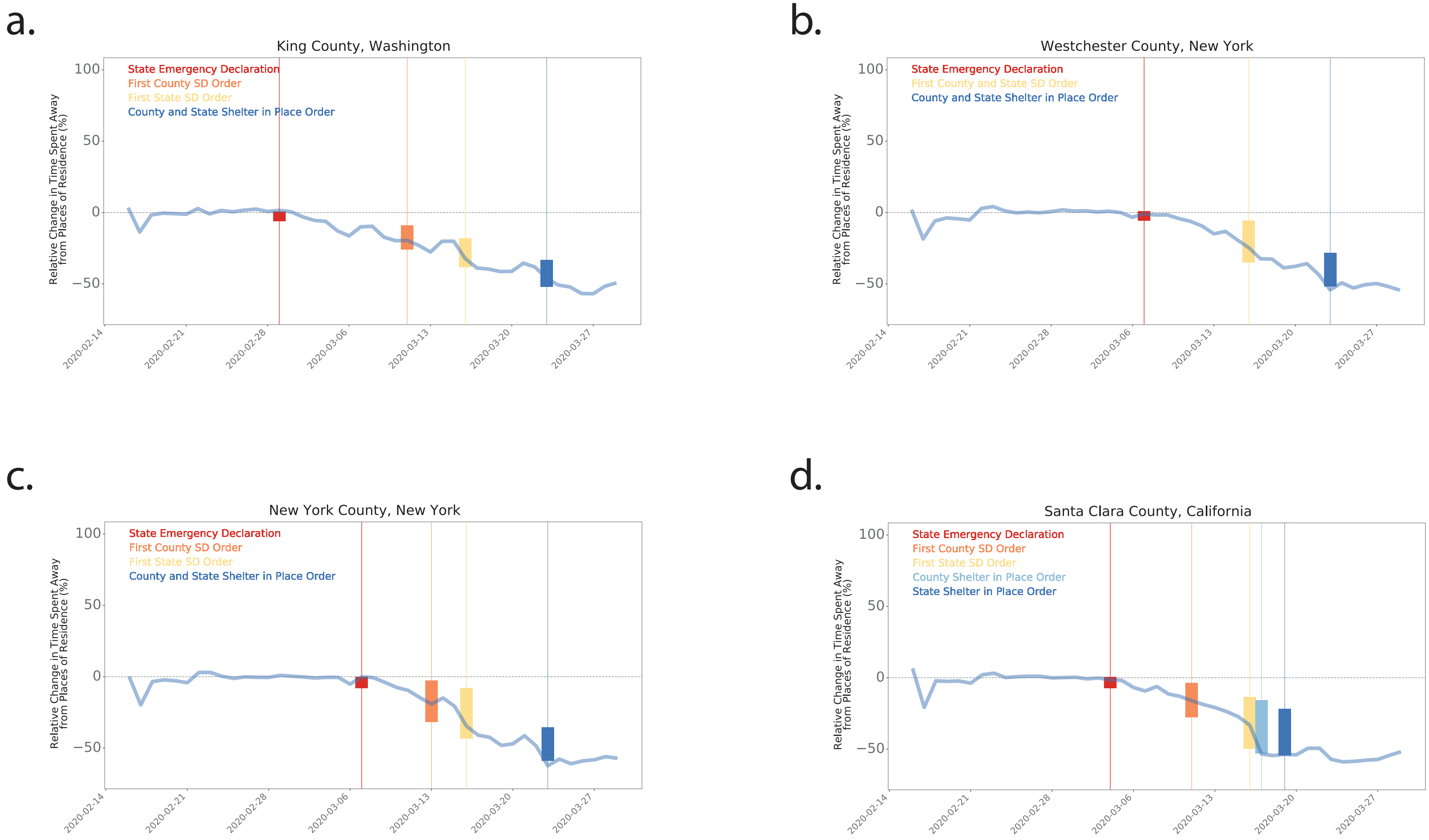

Supplement: Supplementary file 1 — Supplementary Information [file 41467_2021_23404_MOESM1_ESM.pdf]
