# Supplementary material for: Impacts of social distancing policies on mobility and COVID-19 case growth in the US
Source: Nat Commun. 2021 May 25;12:3118. doi: 10.1038/s41467-021-23404-5 (PMC8149701; doi:10.1038/s41467-021-23404-5)
Supplement: Supplementary file 3 — Reporting Summary [file 41467_2021_23404_MOESM3_ESM.pdf]

nature research

Corresponding author(s): Gabrilovich, NMED-L104062

Last updated by author(s): Feb 11, 2021

Reporting Summary

Nature Research wishes to improve the reproducibility of the work that we publish. This form provides structure for consistency and transparency in reporting. For further information on Nature Research policies, see our Editorial Policies and the Editorial Policy Checklist.

Statistics

For all statistical analyses, confirm that the following items are present in the figure legend, table legend, main text, or Methods section.

n/a	Confirmed	
☐	☒	The exact sample size (n) for each experimental group/condition, given as a discrete number and unit of measurement
☐	☒	A statement on whether measurements were taken from distinct samples or whether the same sample was measured repeatedly
☐	☒	The statistical test(s) used AND whether they are one- or two-sided<br>*Only common tests should be described solely by name; describe more complex techniques in the Methods section.*
☐	☒	A description of all covariates tested
☐	☒	A description of any assumptions or corrections, such as tests of normality and adjustment for multiple comparisons
☐	☒	A full description of the statistical parameters including central tendency (e.g. means) or other basic estimates (e.g. regression coefficient) AND variation (e.g. standard deviation) or associated estimates of uncertainty (e.g. confidence intervals)
☐	☒	For null hypothesis testing, the test statistic (e.g. F, t, r) with confidence intervals, effect sizes, degrees of freedom and P value noted<br>*Give P values as exact values whenever suitable.*
☒	☐	For Bayesian analysis, information on the choice of priors and Markov chain Monte Carlo settings
☒	☐	For hierarchical and complex designs, identification of the appropriate level for tests and full reporting of outcomes
☐	☒	Estimates of effect sizes (e.g. Cohen's d, Pearson's r), indicating how they were calculated

Our web collection on statistics for biologists contains articles on many of the points above.

Software and code

Policy information about availability of computer code

Data collection	No new data was collected for this study.
Data analysis	All analyses were performed using python 3.6.7 and graphics were created using the package plotnine 0.6.0.

For manuscripts utilizing custom algorithms or software that are central to the research but not yet described in published literature, software must be made available to editors and reviewers. We strongly encourage code deposition in a community repository (e.g. GitHub). See the Nature Research guidelines for submitting code & software for further information.

Data

Policy information about availability of data

All manuscripts must include a data availability statement. This statement should provide the following information, where applicable:

- Accession codes, unique identifiers, or web links for publicly available datasets
- A list of figures that have associated raw data
- A description of any restrictions on data availability

The anonymized and aggregated dataset analyzed herein was the same one that was used to create the publicly-available Google COVID-19 Community Mobility Reports (first published at http://google.com/covid19/mobility on April 2, 2020). The data analyzed in this paper consisted of anonymized, aggregated, and differentially private counts of visits to places in different categories. The publicly available data reflects ratios computed using these counts.

The information on dates of policy interventions was aggregated from publicly available data including from the Kaiser Family Foundation (https://www.kff.org/health-costs/issue-brief/state-data-and-policy-actions-to-address-coronavirus/ [Accessed 2020-04-2]), the American Enterprise Institute (https://www.aei.org/covid-2019-action-tracker/ [Accessed 2020-04-2]) and the New York Times (https://www.nytimes.com/interactive/2020/us/coronavirus-stay-at-home-order.html.

[accessed 2020-04-02]). Data on COVID-19 cases were obtained from the Johns Hopkins Coronavirus Resource Center (https://coronavirus.jhu.edu/).

Field-specific reporting

Please select the one below that is the best fit for your research. If you are not sure, read the appropriate sections before making your selection.

☐ Life sciences ☒ Behavioural & social sciences ☐ Ecological, evolutionary & environmental sciences

For a reference copy of the document with all sections, see nature.com/documents/nr-reporting-summary-flat.pdf

Behavioural & social sciences study design

All studies must disclose on these points even when the disclosure is negative.

Study description	This is a quantitative analysis of the impacts of state-level social distancing orders on population mobility (as measured by anonymized and aggregated mobility data from users who have opted into Google Location History) and subsequent change in the growth of COVID-19 cases.
Research sample	Mobility data from Google Location History users aggregated for each US county. The unit of analysis is the county-week.
Sampling strategy	We obtained aggregated and anonymized data from a very large population of Google users on mobile devices in all 50 states and Washington, DC who have opted in to having their Location History data stored. Supplementary Figure 2 and Supplementary Figure 3 show that the mobility data included are sufficient to estimate associations between state-level policies and population mobility of the expected magnitude with acceptable precision. Similarly, we used all available data on COVID-19 cases across the US in the relevant time period. Our results suggest that we were able to estimate the impacts of population mobility on case growth with acceptable precision.
Data collection	The mobility data analyzed in the present work are the same data used to create Google's Community Mobility Reports. Geolocation data are securely obtained from the smart phones of individual users who have opted-in to sharing their data with Google. These data are then aggregated and anonymized using a stringent differential privacy algorithm as previously described in detail. The Community Mobility Reports leverage signals such as relative frequency, time, and duration of visits to calculate metrics related to places of residence and places of work of Location History users as described elsewhere. Data collection is automated and independent of research efforts, effectively blinding or separating researchers from data collection. Information on state-level social distancing policies and on COVID-19 case counts were obtained from publicly available sources as described in the manuscript.
Timing	Data were available daily from January 3 through March 29, 2020.
Data exclusions	We note that less populous counties are more likely to have days with missing data for visits to one or more categories of places (e.g., pharmacies) due to privacy filtering and other technical aspects. However, we believe that missing data has negligible effects on the state and national estimates provided, as described in the Supplemental Information in the manuscript.
Non-participation	Data are only available on users that have opted in to Location History.
Randomization	This is a quantitative retrospective analysis using existing data. Randomization of people or populations to different social distancing policies is not appropriate or possible. Randomization of population mobility is not appropriate or possible.

Reporting for specific materials, systems and methods

We require information from authors about some types of materials, experimental systems and methods used in many studies. Here, indicate whether each material, system or method listed is relevant to your study. If you are not sure if a list item applies to your research, read the appropriate section before selecting a response.

Materials & experimental systems

n/a	Involved in the study
☒	☐ Antibodies
☒	☐ Eukaryotic cell lines
☒	☐ Palaeontology and archaeology
☒	☐ Animals and other organisms
☒	☐ Human research participants
☒	☐ Clinical data
☒	☐ Dual use research of concern

Methods

n/a	Involved in the study
☒	☐ ChIP-seq
☒	☐ Flow cytometry
☒	☐ MRI-based neuroimaging
